# Tumor necrosis factor-alpha-308 gene promoter polymorphism associates with survival of cancer patients: A meta-analysis

**DOI:** 10.1097/MD.0000000000013160

**Published:** 2018-11-09

**Authors:** Fengshuang Yi, Xinyu Shi, Xuebin Pei, Xiuzhi Wu

**Affiliations:** a Medical Research Center; b Department of Respiratory and Critical Care Medicine, Beijing Chao-Yang Hospital, Capital Medical University, Beijing, China.

**Keywords:** cancer, gene polymorphism, prognosis, tumor necrosis factor-α

## Abstract

**Background:**

Tumor necrosis factor-alpha (TNF-α) is involved in cancer pathogenesis, and TNF-α-308G>A, a single-nucleotide polymorphism, is associated with cancer prognosis; however, different studies have reported inconsistent results. This meta-analysis aimed to determine the correlation between TNF-α-308G>A polymorphism and the survival of cancer patients.

**Methods:**

PubMed, Web of Science, Embase, Wanfang database, VIP database, and China National Knowledge Infrastructure database were used to obtain articles on association between TNF-α-308G>A polymorphism and cancer survival, published until April 2018. A meta-analysis was carried out using Stata 12.0 software to determine the pooled hazard ratio (HR) and 95% confidence intervals (95% CI). Furthermore, publication bias was assessed, and sensitivity analysis was performed to validate the analysis.

**Results:**

In total, 13 retrospective cohort studies including 2559 cancer patients were reviewed to estimate the association between TNF-α-308G>A polymorphism and overall survival (OS) of cancer patients. The pooled results suggested that within TNF-α-308G>A polymorphism, genotypes GA+AA/GG (HR = 1.39, 95% CI: 0.90–2.14, *P* < .001, I^2^ = 78.1%), GA/GG (HR = 1.06, 95% CI: 0.83–1.36, *P* = .072, I^2^ = 53.5%), and AA/AG+GG (HR = 3.28, 95% CI: 0.92–11.72, *P* = .001, I^2^ = 85.9%) were not associated with the OS of cancer patients. However, interestingly, the HR was greater for patients with the AA genotype than for those with the GG genotype, suggesting an association between TNF-α-308G>A polymorphism and OS among cancer patients (AA/GG, HR = 2.16, 95% CI: 1.36–3.43, *P* = .281, I^2^ = 21.5%).

**Conclusion:**

TNF-α-308G>A polymorphism affects the OS of cancer patients and is a potential therapeutic target for cancer.

## 1 Introduction

Tumor necrosis factor-alpha (TNF-α) was first reported in 1975; it mapped to chromosome 6p21.3 and was cloned in 1985.^[1,2]^ Its gene encodes a 233-amino-acid type II transmembrane protein, which forms stable homotrimers.^[3,4]^ On binding its cognate receptor, many intracellular downstream signaling pathways are activated, including the nuclear factor (NF)-κB, mitogen-activated protein kinase (MAPK), c-Jun N-terminal kinase (JNK), and cell death signaling pathways.^[5–7]^ Furthermore, it functions as a cytokine in numerous aspects of immune regulation.

Genetic epidemiology studies have reported that cytokine polymorphisms are associated with cancer prognosis. Gene polymorphisms in regulatory regions can affect protein expression. A G-to-A substitution in the promoter region (−308) of the TNF-α gene (TNF-α-308G>A) upregulates TNF-α expression and is reportedly associated with many diseases including cancer.^[8–10]^ Although numerous studies have attempted to elucidate the correlation between TNF-α-308G>A polymorphism and cancer risk or prognosis, it is yet controversial whether TNF-α-308G>A polymorphism is a beneficial indicator for overall survival of cancer patients.

This study is the first, to our knowledge, comprehensive meta-analysis of available studies for global analysis of an association between TNF-α-308 polymorphism and overall survival of cancer patients.

## 2 Materials and methods

### 2.1 Review of the literature

We reviewed the literature from PubMed, Web of Science, Embase, Wanfang database, VIP database, and China National Knowledge Infrastructure database, using the search terms “TNF-α,” “−308,” “cancer,” and “survival” to obtain articles published until April 2018, on genotypes of TNF-α-308 polymorphism (rs1800629) in cancer. After excluding duplicate publications and a careful review of the literature, we finally included 13 retrospective cohort studies for our meta-analysis. All analyses in the current study were based on published studies, so no ethical approval and patient consent were required. Data extraction and literature searches were accomplished and double-checked by 2 individuals.

### 2.2 Inclusion criteria

The inclusion criteria for articles were as follows: the use of histological analysis to diagnose and confirm the cancer; evaluation of the correlation between TNF-α-308 polymorphism and overall survival of cancer patients, with an available hazard ratio (HR) and the corresponding 95% confidence interval (95% CI); articles written in English or Chinese; genotyping analysis performed for DNA; studies including exclusively adult subjects without immune diseases. Among studies with overlapping patients, the larger or more comprehensive study would be selected.

### 2.3 Data extraction

Initially, we first screened the title and abstract, and then reviewed the full text carefully. Related data were extracted from each eligible study by 2 investigators (FY and XS), independently, including the first author, year of publication, study location, ethnicity of the study populations, number of genotypes, cancer type, genotyping method, HR, and the corresponding 95% CI for overall survival.

### 2.4 Statistical analysis

We extracted the HRs and the corresponding 95% CIs for overall survival (OS) either directly from the primary reports or calculated them from the OS curves, using a previously reported method.^[11–13]^ Overall survival curves in the included articles were imported into Engauge Digitizer version 4.1 (https://sourceforge.net/projects/digitizer/); thereafter, HRs and the corresponding 95% CIs were calculated as reported previously.^[14]^ An I^2^ value less than 25% and *P* > .05 indicated no significant heterogeneity among the articles. Publication bias was analyzed using Begg and Egger tests for all genetic models.^[15,16]^ All statistical tests were performed using Stata version 12.0 (StataCrop, College Station, TX).

## 3 Results

### 3.1 Inclusion of eligible studies

Upon reviewing the literature and excluding duplicate publications, we obtained 13 full-text reviews on the basis of our strict screening criteria (Table 1). Among these, 3 reviews were based on breast cancer,^[7,17,18]^ 2 were on esophageal squamous cell carcinoma,^[19,20]^ and the remaining reviews were on pancreatic cancer, bladder cancer, gastro-oesophageal cancer, non-Hodgkin lymphoma, head and neck squamous cell carcinoma, nonsmall cell lung cancer, diffuse B-cell lymphomas, and gastric cancer.^[9,21–27]^

**Table 1:**
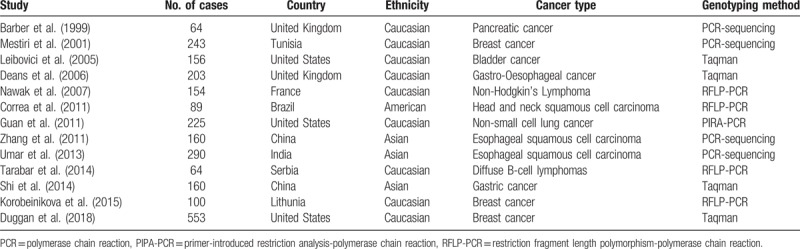
Characteristics of eligible studies in this meta-analysis.

### 3.2 Overall survival of cancer patients harboring the TNF-α-308G>A polymorphism

**Figure 1:**
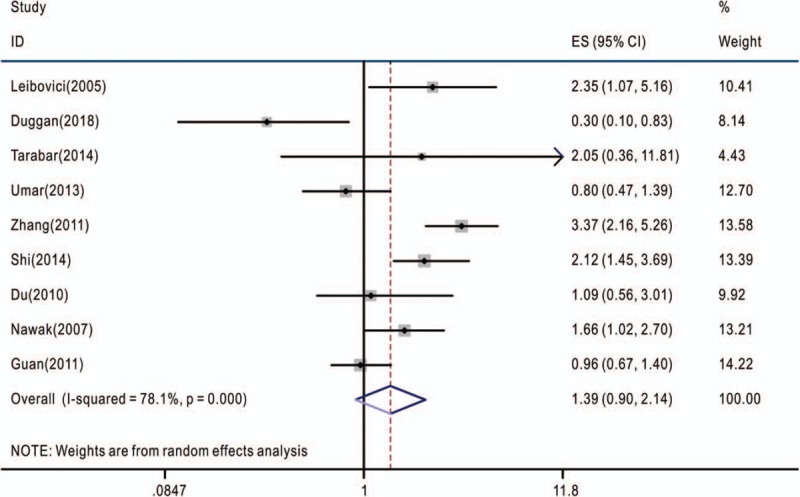
Overall association between TNF-α-308G>A polymorphism and the overall survival of cancer patients (genotype GA+AA/GG). The hazard ratios (HRs) in the studies and the corresponding 95% confidence intervals (CIs) are plotted with horizontal lines. Pooled HR and the corresponding 95% CI values are denoted by the diamond symbol. TNF-α = tumor necrosis factor-alpha.

**Figure 2:**
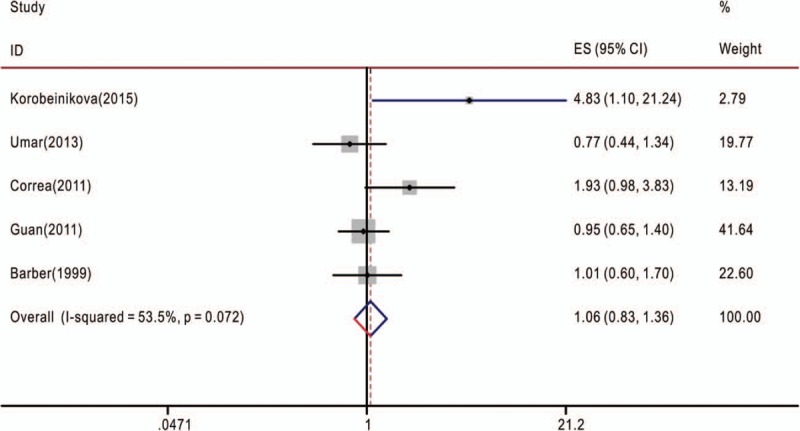
Overall association between TNF-α-308G>A polymorphism and the overall survival of cancer patients (genotype GA/GG). The hazard ratios (HRs) in the studies and the corresponding 95% confidence intervals (CIs) are plotted with horizontal lines. Pooled HR and the corresponding 95% CI values are denoted by the diamond symbol. TNF-α = tumor necrosis factor-alpha.

**Figure 3:**
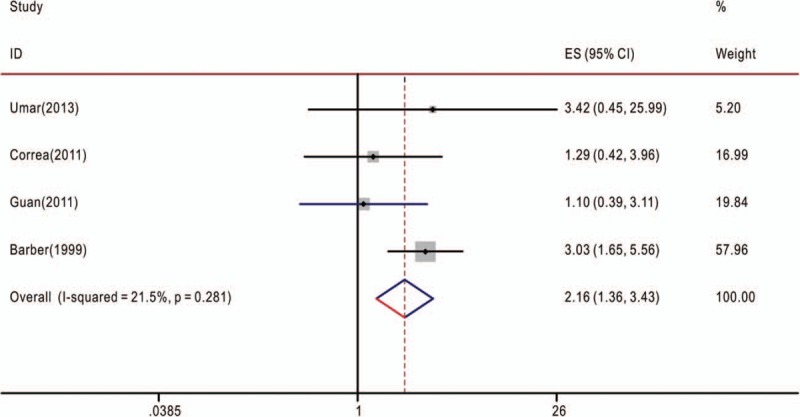
Overall association between TNF-α-308G>A polymorphism and the overall survival of cancer patients (genotype AA/GG). The hazard ratios (HRs) in the studies and the corresponding 95% confidence intervals (CIs) are plotted with horizontal lines. Pooled HR and the corresponding 95% CI values are denoted by the diamond symbol. TNF-α = tumor necrosis factor-alpha.

**Figure 4:**
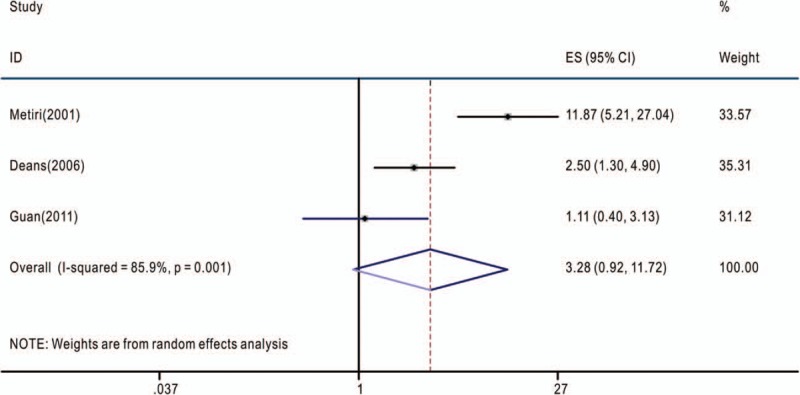
Overall association between TNF-α-308G>A polymorphism and the overall survival of cancer patients (genotype AA/GA+GG). The hazard ratios (HRs) in the studies and the corresponding 95% confidence intervals (CIs) are plotted with horizontal lines. Pooled HR and the corresponding 95% CI values are denoted by the diamond symbol. TNF-α = tumor necrosis factor-alpha.

The associations between TNF-α-308G>A polymorphism and OS of cancer patients in each eligible study are presented in Table 2. We assessed the combined data from 13 studies including 2559 patients, using 4 genetic models, they are GA+AA/GG, GA/GG, AA/GG, and AA/AG+GG. GA+AA/GG represents comparison between patients who carry TNF-α-308 GA or AA genotypes and who carry TNF-α-308 GG genotypes; GA/GG represents comparison between patients who carry TNF-α-308 GA genotypes and who carry TNF-α-308 GG genotypes; AA/GG represents comparison between patients who carry TNF-α-308 AA genotypes and who carry TNF-α-308 GG genotypes; AA/AG+GG represents comparison between patients who carry TNF-α-308 AA genotypes and who carry TNF-α-308 AG or GG genotypes. Compared with the GG phenotype, the TNF-α-308 GA, and AA genotypes were not associated with OS (GA+AA/GG, HR = 1.39, 95% CI: 0.90–2.14, *P* < .001, I^2^ = 78.1%, random effects model) (Fig. 1), and similar results were obtained with the TNF-α-308 GA genotype, compared with the GG genotype (GA/GG, HR = 1.06, 95% CI: 0.83–1.36, *P* = .072, I^2^ = 53.5%) (Fig. 2), suggesting that TNF-α-308 GA genotype was also not associated with OS, compared with the GG genotype. However, interestingly, upon comparative analysis of the data on the TNF-α-308 AA and GG genotypes, the AA genotype was associated with a low overall survival rate of cancer patients, compared with patients with the GG genotype (AA/GG). The pooled HR was 2.16, 95% CI: 1.36 to 3.43, *P* = .281, I^2^ = 21.5% (Fig. 3). Only 3 studies analyzed the association of the TNF-α-308 AA genotype with OS, compared with the AG and GG genotypes, the pooled results regarding the obvious heterogeneity may not be very reliable (AA/AG+GG, HR = 3.28, 95% CI: 0.92–11.72, *P* = .001, I^2^ = 85.9%, random effects model) (Fig. 4). For GA+AA/GG genotype, sensitivity analysis revealed that the pooled HR and its 95% CI estimated using a random effects model were different from that estimated using a fixed effects model (GA+AA/GG, HR = 1.48, 95% CI: 1.23–1.78, *P* < .001, I^2^ = 85.9%, fixed effects model). For the AA/AG+GG genotype, sensitivity analysis revealed that the pooled HR and its 95% CI estimated using the random effects model were significantly different from those estimated using a fixed effects model (AA/AG+GG, HR = 3.46, 95% CI: 2.18–5.5, *P* = .001, I^2^ = 85.9%, fixed effects model). Except for genotypes GA+AA/GG and AA/AG+GG, sensitivity analysis for the other 2 genotypes revealed a slight but not significant difference between the random and the fixed effects models, thereby highlighting the reliability of our meta-analysis.

**Table 2:**
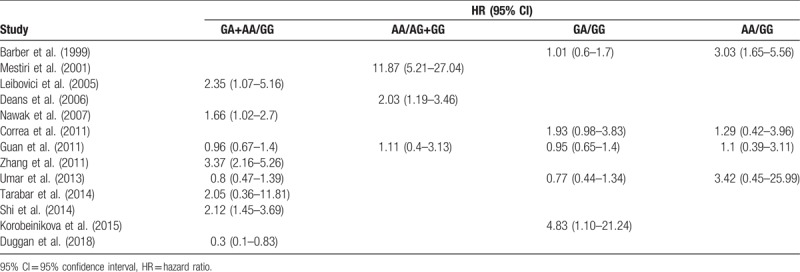
Summary of primary data for association between TNF-α-308G>A polymorphism and overall survival of cancer from 13 eligible studies.

### 3.3 Sensitivity analysis and publication bias

Sensitivity analysis performed using fixed and random effects models exhibited a slight but not significant difference between genotypes GA/GG and AA/GG; however, sensitivity analysis including fixed and random effects model analyses of genotypes GA+AA/GG and AA/AG+GG exhibited marked differences between these genotypes, thereby highlighting the reliability of our meta-analysis for the former 2 genotypes. We also evaluated the publication bias of included studies for each model, using Begg and Egger tests. No evidence of obvious asymmetry was obtained in overall analysis of publication bias for TNF-α-308G>A polymorphism in each genetic model.

## 4 Discussion

TNF-α was initially considered to be produced primarily by macrophages and other cells including lymphoid cells, mast cells, endothelial cells, cardiac myocytes and adipose tissue, fibroblasts, and neurons.^[28]^ Dysregulation of TNF-α production is reportedly implicated in various human diseases including Alzheimer disease, major depression, psoriasis, inflammatory bowel disease, and cancer.^[10,29]^ Increasing evidence indicates that genetic polymorphisms in the promoter region of TNF-α influences its translation and corresponding malignancies.^[30]^ The TNF-α-308G>A polymorphic site is one of the most well-defined polymorphisms, wherein GG is the wild genotype and the A allele is associated with TNF-α up regulation and poor clinical outcomes among cancer patients.^[8,18,31,32]^ However, certain studies have also reported the absence of an association between TNF-α-308G>A polymorphism genotypes and breast cancer,^[33,34]^ although several other studies have suggested an association between TNF-α-308G>A polymorphism and breast cancer risk.^[35,36]^

The present meta-analysis involved the extraction of data strictly based on the defined criteria and grouped the related studies into 4 subgroups in accordance with the reported genotypes: GA+AA/GG, GA/GG, AA/GG, and AA/AG+GG. Overall, our results suggest that only the AA/GG genotype essentially predicted cancer patient survival; patients harboring the AA genotype at TNF-α-308 had a shorter lifespan compared to those harboring the GG genotype, with no obvious heterogeneity (Fig. 3). Genotypes GA+AA/GG and GA/GG were not associated with the OS of cancer patients, although some studies reported that these genotypes had adverse or protective effects on patients with breast cancer, gastric cancer, bladder cancer, or esophageal squamous cell carcinoma^[7,9,18,20,27]^ (Figs. 1 and 2). Furthermore, genotype AA/AG+GG was not associated with the OS of cancer patients; the obvious heterogeneity and differences in the results of sensitivity analysis via fixed and random effects models rendered this genotype unreliable owing to its small cohort. We also tried to analyze the association between TNF-α-308 genotypes and OS of patients with specific types of cancer. But none of the 4 genetic models included more than 2 reports with 1 specific cancer type (Tables 1 and 2), so more work should be done in this field in the future.

Despite this study being the first comprehensive meta-analysis on the association between TNF-α-308G>A polymorphism and cancer prognosis, this meta-analysis has some limitations. First, the number of eligible articles and patients was relatively small; hence, the 4 different genotype models should be estimated separately. Moreover, cancer prognosis is very complicated and is associated with numerous factors. For example, some studies have estimated the effects of certain treatments on the therapeutic outcomes of cancer patients, while others have not. Studies not highlighting these effects are indistinguishable; hence, we analyzed them collectively. Moreover, TNF-α-308G>A polymorphism is the only single-nucleotide polymorphism we estimated in the current study; however, others including −238G>A and −857C>T were not included.^[37,38]^

In conclusion, our study suggests the prediction role of TNF-α-308G>A polymorphism in cancer patient survival. However, owing to limitations in the sample size, future studies are required to assess the association between genotypes including TNF-α-308G>A polymorphism and clinical cancer prognosis.

## Author contributions

**Conceptualization:** Fengshuang Yi, Xiuzhi Wu.

**Data curation:** Fengshuang Yi, Xinyu Shi, Xuebin Pei, Xiuzhi Wu.

**Formal analysis:** Fengshuang Yi, Xinyu Shi, Xuebin Pei.

**Funding acquisition:** Fengshuang Yi.

**Investigation:** Fengshuang Yi.

**Methodology:** Fengshuang Yi, Xinyu Shi, Xuebin Pei.

**Project administration:** Fengshuang Yi, Xuebin Pei, Xiuzhi Wu.

**Resources:** Fengshuang Yi, Xuebin Pei, Xiuzhi Wu.

**Software:** Fengshuang Yi, Xinyu Shi.

**Supervision:** Fengshuang Yi.

**Validation:** Fengshuang Yi, Xinyu Shi, Xuebin Pei, Xiuzhi Wu.

**Visualization:** Fengshuang Yi, Xiuzhi Wu.

**Writing – original draft:** Fengshuang Yi, Xinyu Shi, Xuebin Pei, Xiuzhi Wu.

**Writing – review & editing:** Fengshuang Yi, Xiuzhi Wu.
